# Enhancement of the Physical and Functional Properties of Chitosan Films by Incorporating *Galla chinensis* Extract

**DOI:** 10.3390/antiox13010069

**Published:** 2024-01-02

**Authors:** Ran Tao, Xiuxia Zheng, Bei Fan, Xuemei He, Jian Sun, Yufeng Sun, Fengzhong Wang

**Affiliations:** 1 Institute of Food Science and Technology, Chinese Academy of Agricultural Sciences, Beijing 100193, China18646654981@163.com (X.Z.);; 2 Guangxi Academy of Agricultural Sciences, Nanning 530007, Chinajiansun@gxaas.net (J.S.)

**Keywords:** chitosan, *Galla chinensis* extract, physical property, functional, antioxidant, fruit preservation

## Abstract

Composite films based on chitosan (CS) incorporating *Galla chinensis* extract (GCNE) at different CS/GCNE weight ratios, which are both biodegradable and multifunctional, were fabricated using the solution-casting method. The FTIR analyses indicated that a good interaction was presented among the GCNE and CS through an intermolecular hydrogen bond. The incorporation of the GCNE improved the films’ elongation at break, UV-light blocking, and decreased the moisture regain (from 16.68% to 10.69%) and water absorption (from 80.65% to 54.74%). Moreover, the CS/GCNE films exhibited a strong antioxidant activity (from 57.11% to 70.37% of DPPH and from 35.53% to 46.73% of ABTS scavenging activities) mainly due to the high content of phenolic compounds in the incorporated GCNE. The CS/GCNE film-forming solution coatings demonstrated their effectiveness in preserving the quality of postharvest mangoes, specifically by minimizing the change in the firmness, weight loss, titratable acidity, and total phenolic and ascorbic acids. These findings suggest that the multifunctional composite films possess a high application potential to preserve postharvest fruits.

## 1. Introduction

Fresh fruits are indispensable elements of the human daily diet, providing diverse vitamins and nutrients for consumers. Fresh fruits are welcomed, not only due to their health-promoting properties, but also desirable taste and flavor. Postharvest fruits still maintain respiration and ripening processes. They are susceptible to the loss of sensory and nutritional characteristics [1], resulting in the reduction in consumer acceptance, the shortness of shelf-life, and economic loss [2]. In the past decades, to minimize the quality loss of postharvest fruits, various treatments have been applied. Packaging was used to provide protection from hazards and to increase the shelf-life of fruit. Recently, the nondegradable materials have caused extensive white pollution [3], which has sparked the public’s desire for environmentally friendly packaging [4].

Chitosan (CS), obtained from naturally occurring chitin after the deacetylation process, is the most popular biodegradable biopolymer used for the formation of packaging materials, owing to its excellent film-forming properties, biocompatibility, edibility, and abundance [5,6,7]. CS-based films and coatings are stable systems used as carriers of active ingredients, which have received more attention. However, CS has a low antioxidant activity. Song et al. developed a CS–magnolol film, which had a high antioxidant activity and low-water-vapor permeability, and could be effectively applied to pork preservation [8]. Yadav et al. synthesized chitosan–gelatin biocomposite films containing quercetin–starch, which showed enhanced antioxidant activity and barrier properties against UV rays and water vapor, promising its application in food packaging [9]. Perdones et al. fabricated CS coatings for controlling strawberry decay at 20 °C over a 7-day period using lemon essential oils. These CS coatings exhibited remarkable effectiveness. Nonetheless, it is worth mentioning that the application of these coatings adversely affected the aroma profile of the fruits [10].

*Galla chinensis*, a traditional Chinese herb, is the gall of the Rhus leaf generated from abnormal growth in response to parasitic aphids, *Melaphis chinensis* (Bell) Baker [11]. *G. chinensis* contains abundant phenolic compounds, such as tannins, gallic acid, and methyl gallate. It has antioxidant, antimicrobial, anti-inflammatory activity, etc. [12]. Tannins from *G. chinensis* have been reported as functional components to prepare CS-based composite fibers to decrease the hydrophilicity and increase the breaking strength and bacterial reduction to Staphylococcus aureus [13]. It can also be loaded onto chitosan/MXene (or starch and gelatin)-based films to improve the preservation of fruit [14,15,16]. Gallic acid has been demonstrated to remarkably elevate the antioxidant and tensile strength potential of CS, thereby broadening the development of CS-based films [17]. The practical application of CS-based packaging material by incorporating monomer active ingredients is limited due to the uneconomical extraction procedure of monomer active ingredients from organisms. However, plant extracts, which have been gaining more interest among researchers because of their low cost and functionality, could be used as an alternative for monomer active ingredients.

The objective of this study was to develop biodegradable composite films based on CS, incorporating *G. chinensis* extract (GCNE), which has a low odor. The microstructures of the films were observed by scanning electron microscopy (SEM) and Fourier transform infrared spectroscopy (FTIR). The films were characterized with regard to the mechanical properties, color, light transmission, moisture regain (MR), water absorption (WA), water vapor permeability (WVP), and their application for mango preservation. Overall, this work will provide an economical, environmentally friendly, and multifunctional packaging material for postharvest fruit preservation.

## 2. Materials and Methods

### 2.1. Materials

*G. chinensis* was obtained from TongRenTang Medicine (Beijing, China). CS (M_w_: 375 kDa, DD: 95%) was purchased from Macklin (Shanghai, China). Glycerol (with a purity of 99.0%) and acetic acid solution (1 N) were obtained from Sinopharm (Beijing, China). Folin–Ciocalteu reagent (1 M) and 2,2-diphenyl-1-picrylhydrazyl (DPPH) were purchased from Sangon (Shanghai, China). The ABTS free-radical scavenging ability test kit was obtained from Solarbio (Beijing, China). Deionized water was prepared by the Heal Force system (Shanghai Likang, Instruments Co., Ltd., Shanghai, China). All other reagents were of analytical grade and obtained from Yuanye (Shanghai, China). Mango (*Mangifera indica* L.) was collected from a local farmers market (Beijing, China). The mangoes used for the treatment exhibited a consistent size, shape, and color, and were devoid of any impairment or deterioration.

### 2.2. Preparation and Characterization of the GCNE

*G. chinensis* powder (60 mesh) was added to 80% ethanol with a material–liquid ratio of 1:10 (g/mL). After soaking for 1 h, the system was ultrasonically treated at 76 °C for 30 min. After centrifugation (8000 rpm), the supernatant was concentrated to afford the GCNE.

The total phenolic content of the GCNE was measured by the Folin–Ciocalteu method. Briefly, the UV–vis absorbance standard curve of the gallic acid was firstly established using a UV–vis spectrophotometer (UV-8000S, Shanghai Metash Instruments Co., Ltd., Shanghai, China). Then, the GCNE solution (0.1 mg/mL, 0.5 mL) was added to the Folin–Ciocalteu reagent (1 mL). After mixing for 5 min, and the addition of a sodium carbonate solution (7.5%, 1 mL), the mixture was placed in the dark for 1 h. The absorbance of the mixture was measured. The total phenolic content was expressed by gallic acid. Therefore, the total phenolic content was calculated according to the gallic acid standard curve.

The gallic acid content of the GCNE was determined by LC-MS/MS (Waters Nexera X2 UPLC and Waters 4500 TQXS MS/MS, Waters, MA, USA). Specifically, the ACOUITY UPLC BEH C18 column (2.1 × 100 mm, 1.7 μm, Waters) was utilized for conducting chromatographic separations at 35 °C. The mobile phases comprised two solvents, A (water with 0.1% formic acid) and B (acetonitrile). This mobile phase system was run as follows: 0–5% B (0–0.5 min), 5–95% B (0.5–3 min), 95–95% B (3–4 min), 95–5% B (4–5 min), and 5–5% B (5–6 min). The injection volume was 10 μL, while the flow rate was set at 300 μL/min. The mass spectrometry conditions were as follows: negative-ion and MRM mode, sheath gas, 30 psi; capillary voltage, 4000 V; drying gas temperature, 3000 °C; drying gas flow rate, 8 L/min.

### 2.3. Preparation of the Films

The films were fabricated by the solution-casting method [18]. CS powder (3 g) was added to the acetic acid solution (1%, 200 mL) with stirring until the particles were thoroughly dispersed. Glycerol (0.45 g) was added as a plasticizer. The resulting solution was stirred for 10 min at 40 °C. Then, 0, 10, 20, and 30 wt% (based on the CS weight) of the GCNE were added to the mixture, respectively. The systems were then stirred for 1 h at 40 °C and ultrasonicated for 10 min at 40 °C. An amount of 20 g from each solution was added into Petri dishes (9 cm in diameter) and dried at 55 °C for 4 h. All the obtained films (CS, CS-GCNE 10%, CS-GCNE 20%, and CS-GCNE 30%) were stored at 23 °C and 45% RH for further tests.

### 2.4. Structural Characterization of the Films

The internal morphologies of the films were analyzed by SEM (SU8010, Hitachi, TYO, Japan). The samples were gold-coated in a sputtering unit and measured at a 10 kV working voltage. The FTIR spectra of the films were evaluated using a TENSOR FTIR spectrometer (Bruker, SB, German) by an attenuated total reflection method in a 4 cm^−1^ step size from 4000 to 500 cm^−1^, with an average of 70 scans.

### 2.5. Mechanical Properties of the Films

The mechanical properties of the films, including the tensile strength (TS) and elongation at break (EB), were measured as described previously [19]. The film strips (1 × 7 cm) were tested using a physical property tester (TA.HD plus, Stable Micro Systems, LND, UK) with an initial grip separation of 20 mm and a crosshead speed of 50 mm/min. Each group of films was repeatedly determined 4 times. The TS and EB were calculated as followed:

(1) $$TS=\frac{F}{x\times W}$$

where F (N) represents the maximum tension experienced during membrane rupture, x (mm) represents the thickness of the film (determined by a hand-held micrometer at 5 points randomly: CS film: 0.079 ± 0.002; CS-GCNE 10% film: 0.078 ± 0.002; CS-GCNE 20% film: 0.078 ± 0.001; CS-GCNE 30% film: 0.076 ± 0.002), and W (mm) represents the width of the film.

(2) $$EB\left(\%\right)=\frac{D-D_{0}}{D_{0}}\times 100$$

where D (mm) is distance between the markings during the membrane rupture and D_0_ (mm) is the original marking distance of the membrane.

### 2.6. Determination of the Physical Properties of the Films

An electronic eye (Digieye DigitalImaging System, VeriVide, LND, UK) was used for measuring the surface color of the films. Before the measurement, calibration was carried out with a standard white plate. The total color difference (ΔE) was calculated with an equation from Rhim [20].

(3) $$\Delta E=\sqrt{{\left(L-L_{0}\right)}^{2}+{\left(a-a_{0}\right)}^{2}+{\left(b-b_{0}\right)}^{2}}$$

where L_0_, a_0_, and b_0_ are the standard values of a white plate, and L, a, and b are the measured color profile values of the film samples. Each sample was tested in triplicate. Standard values for the white calibration plate were L_0_ = 97.39, a_0_ = 0.03, and b_0_ = 1.77.

The light transmittances of the films were measured using a UV–vis spectrophotometer. An empty cuvette was employed as the reference. Subsequently, the film samples were then placed into the cuvette in a manner perpendicular to the orientation of the light source. Data acquisition took place across the wavelength range of 200–800 nm.

The MR of the films was determined as described previously [21]. The film strips (3 × 4 cm) were dried at 105 °C for 24 h and weighed (W_1_), then equilibrated at 21 °C and 65% RH for 48 h and weighed (W_2_). The MR of the films was calculated as follows:

(4) $$MR\left(\%\right)=\frac{W_{2}-W_{1}}{W_{2}}\times 100$$

The WA of the films was determined according to previous research [21]. The film strips (3 × 4 cm) were dried at 105 °C for 24 h and weighed (W_3_), then immersed in deionized water (25 mL) at 25 °C for 10 min, centrifuged at 5000 rpm for another 10 min to remove the excess water, and weighed (W_4_). The WA of the films was calculated as follows:

(5) $$WA\left(\%\right)=\frac{W_{4}-W_{3}}{W_{4}}\times 100$$

The WVP of the films was determined by a standard method [22]. The films were fixed horizontally on measuring cups, which contained deionized water (20 mL) under the films. The measuring cups were stored in desiccators at 20 °C and weighed once every 2 h, totaling 6 times. The WVP of the films was calculated as follows:

(6) $$WVP=\frac{x\times ∆m}{t\times ∆P\times S}$$

where x (m), ∆m (g), t (s), ΔP (2.945 kPa), and S (m^2^) represent the thickness of the film, the weight difference, the interval time, the vapor pressure difference between the inside and outside of the cups, and the effective permeable area, respectively.

### 2.7. Antioxidant Activity of the Films

The antioxidant activity of the films was expressed by the DPPH and ABTS radical scavenging activity, and evaluated according to previous research, with some modifications [23]. Five film strips (2 × 2 cm) were immersed in methanol (4 mL) in the dark for 3 h. The solution (3 mL) was reacted with the DPPH methanol solution (150 µmol/L, 1 mL) in the dark for 30 min. Methanol was used as a blank background, and the mixture of the methanol (3 mL) and DPPH methanol solution (150 µmol/L, 1 mL) was used as the reference. The absorbance of the sample solution at 517 nm was measured, and the DPPH radical scavenging activity was calculated as Formula (7). The methanol solution obtained by immersing the film strips was also used to test the ABTS radical scavenging activity according to the kit instructions. The absorbance of the sample solution at 405 nm was measured, and the ABTS radical scavenging activity was calculated as Formula (8).

(7) $$DPPH\;radical\;scavenging\;activity\left(\%\right)=\frac{A_{1}-A_{2}}{A_{1}}\times 100$$

where A_1_ represents the absorbance of the reference and A_2_ represents the absorbance of the test sample.

(8) $$ABTS\;radical\;scavenging\;activity\left(\%\right)=\frac{{A_{0}-(A}_{t}-A_{c})}{A_{0}}\times 100$$

where A_0_ represents the absorbance of the blank (distilled water), A_t_ represents the absorbance of the test sample under test-treated conditions, and A_c_ represents the absorbance of test sample under control-treated conditions.

### 2.8. Mango Preservation Application

The mangoes with identical maturity and size were randomly divided into three groups, and immersed in the film-forming solutions for 2 min, respectively. All the mangoes were air-dried until there was no solvent on the peels and stored (20 °C, 50% RH) for 18 d. During storage, the mango qualities were evaluated every three days in terms of firmness, weight loss, titratable acidity, total phenolic, ascorbic acid, and antioxidant activity.

The firmness of the mangoes was expressed by the maximum penetration pressure achieved during the tissue rupture. It was determined by a hand-held penetrometer (GY-2, Aipli Co., Ltd., Beijing, China).

The weight loss of the mangoes was expressed by the change of weight every three days. It was calculated as follows:

(9) $$W\left(\%\right)=\frac{W_{5}-W_{6}}{W_{5}}\times 100$$

where W_5_ (g) and W_6_ (g) represents the initial weight and the weight after every three days, respectively.

The titratable acidity content of the mangoes was determined by sodium hydroxide titration [24]. Mango pulp (10 g) was added into deionized water (90 mL). After homogeneity and centrifugation, the supernatant was titrated by a 0.1 M sodium hydroxide solution. The results were represented as percentages.

The ascorbic acid content of the mangoes was determined as described previously [25]. Mango pulp (10 g) was added into oxalic acid (2%, 50 mL). After the homogenate and filtration, the filtrate was titrated with a 0.1 M 2,6-dichlorophenol-indophenol solution.

The determination of the total phenolic content of the mangoes was carried out as described above for the determination of the GCNE, and the determination of the antioxidant activity of the mangoes was carried out as described above for the determination of the films. Mango pulp (1 g) was then added into methanol (10 mL). After homogeneity and ultrasound extraction, the obtained solution was used for the test.

### 2.9. Statistical Analysis

The data were analyzed by the analysis of variance with SPSS software 13.0 (SPSS Inc., Chicago, IL, USA). Multiple comparisons among the different systems were conducted using Tukey’s test, considering a statistical significance at the *p* < 0.05 level.

## 3. Results and Discussion

### 3.1. Properties of the GCNE

**Figure 1:**
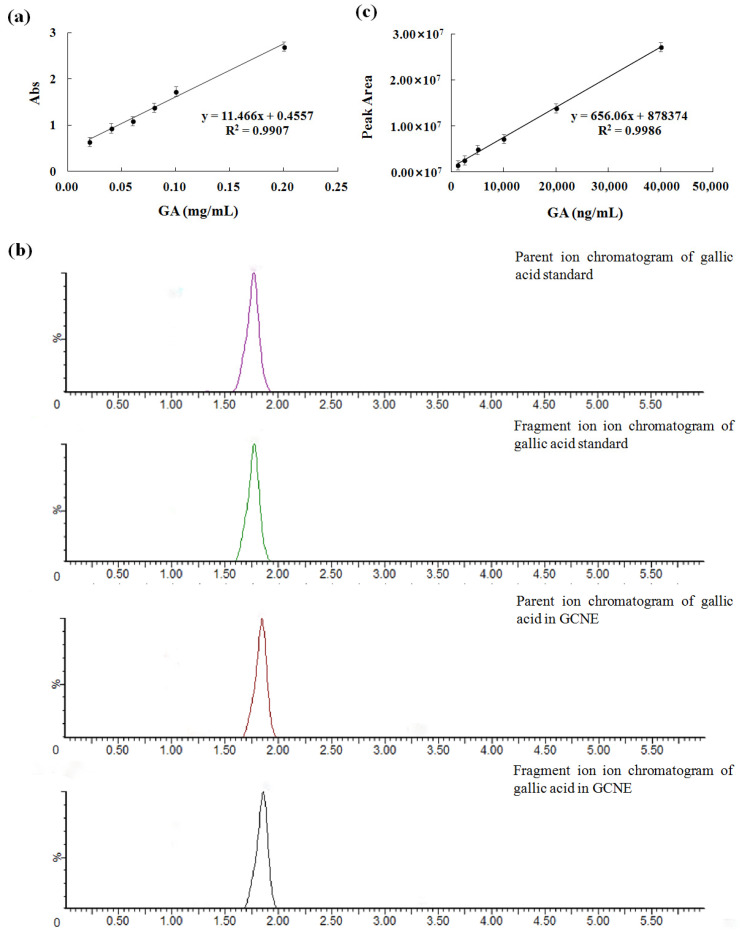
(**a**) UV–vis absorbance standard curve of gallic acid, (**b**) ion chromatograms of the gallic acid in standard and the GCNE, and (**c**) the LC-MS/MS standard curve of the gallic acid.

The GCNE has many beneficial effects, such as protecting rice by inhibiting the mycelial growth of rice sheath blight [26], preparing functionalized few-layer graphene–silver nanocomposites [27], and dying cotton, silk, and wool fabrics [28]. In this work, the GCNE was prepared by the ultrasonic method with a yield of 55.4%. To determine the total phenolic content of the GCNE, the gallic acid standard curve was established, as shown in Figure 1a (correlation coefficient R^2^ = 0.9907). The total phenolic content of the GCNE was determined as 659.92 ± 1.47 mg/g. To determine the gallic acid content of the GCNE, the LC-MS/MS method was established. The ion chromatograms of the gallic acid in the standard and the GCNE are shown in Figure 1b. The peak shape and separation for the gallic acid were excellent. The parent ion (*m*/*z* 125) of the gallic acid standard was chosen as a quantitative ion, and the standard curve was established (Figure 1c). The gallic acid content of the GCNE was determined as 292.15 ± 5.19 mg/g. The high content of total phenolic and gallic acids contributed greatly to the GCNE function.

### 3.2. Internal Morphological Property of the Films

**Figure 2:**
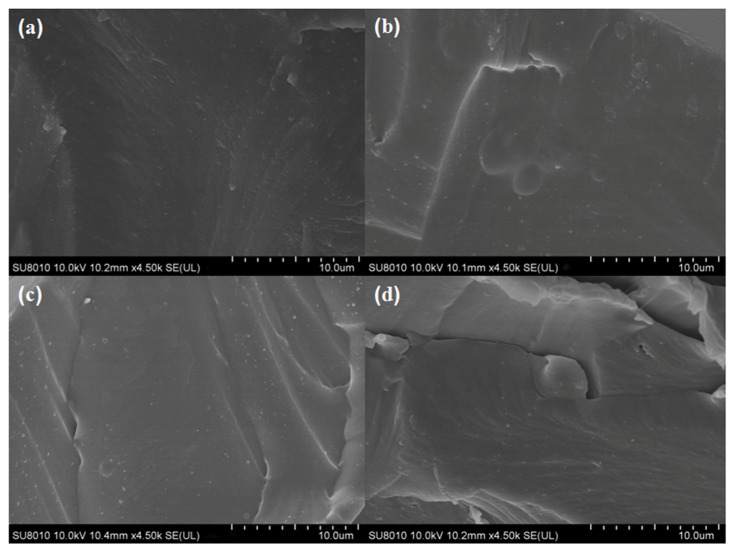
Cross-section SEM images of (**a**) the neat CS film, (**b**) the CS-GCNE 10% film, (**c**) the CS-GCNE 20% film, and (**d**) the CS-GCNE 30% film.

The morphologies of the films were related close to the components’ dispersion state and interactions [29]. As shown in Figure 2a, the internal morphology of the cross-section of the CS film was smooth and dense without any cracks, indicating that the compatibility between the components (e.g., the CS and glycerol) was good. Slight differences between the neat CS and the different CS-GCNE composite films (CS-GCNE 10%, CS-GCNE 20%, and CS-GCNE 30% films) could be found (Figure 2b–d). With the increase of the GCNE content, the internal morphology of the cross-sections of the CS-GCNE films appeared to be almost smooth, with the appearance of some wrinkles that could possibly have resulted from the intense interactions between the CS chains and the GCNE phenolic compounds. These interactions potentially induced the creation of a localized microphase separation within the CS matrix. Similar internal morphologies of the films were also observed in previous research [19].

### 3.3. FTIR Analysis of the Films

**Figure 3:**
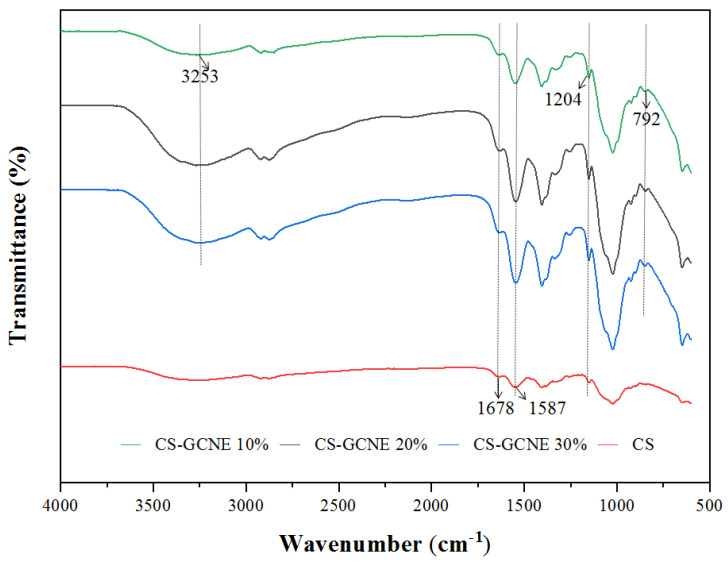
FTIR spectra of the neat CS and CS-GCNE composite films.

The potential interactions between the CS and GCNE were investigated by FTIR spectroscopy. The FTIR spectra of the films are shown in Figure 3. The FTIR spectrum of the neat CS film showed characteristic absorption peaks at 1678 cm^−1^, corresponding to the C=O stretching vibration of N-acetyl residues (amide I), and 1587 cm^−1^, assigned to the N-H bending vibration of the primary amine (amide II) [30]. Slight changes in the FTIR spectrum of the CS film were induced with the GCNE addition. The intensity of the above two characteristic absorption peaks increased with the increasing GCNE content, indicating that intermolecular hydrogen bonding between CS and GCNE had been formed. The FTIR spectrum of the CS-GCNE 10% film showed two characteristic absorption peaks from the added GCNE: a broad absorption peak at 3253 cm^−1^,corresponding to the O-H stretching vibration, and a strong absorption peak at 1204 cm^−1^, corresponding to the tensile pattern of the C-H and polyol C-O [15]. The intensity of the two characteristic absorption peaks also increased with the increasing GCNE content and slightly shifted towards lower frequencies. The band at 792 cm^−1^ related to the C–H out-of-plane bending vibrations of the substitute aromatic ring of the phenolic compounds appeared in the CS-GCNE composite films, indicating that the interaction between the GCNE and CS matrix presented in the resulting films [31].

### 3.4. Mechanical Properties of the Films

**Figure 4:**
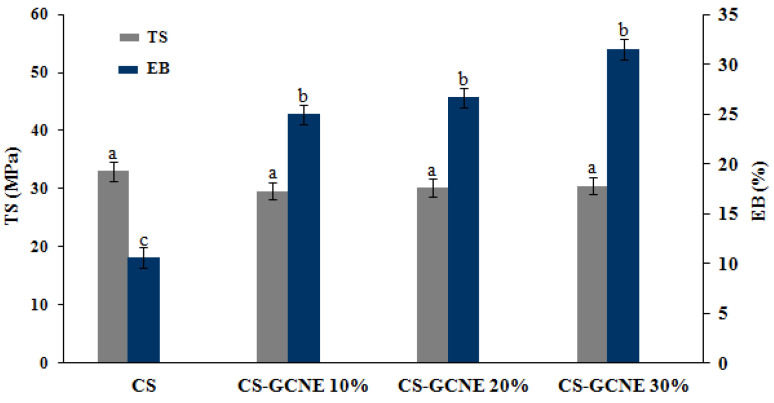
Tensile strength (TS) and elongation at break (EB) of the neat CS and CS-GCNE composite films. Mean values and standard deviation. a–c: different letters indicate significant differences among the films (*p* < 0.05).

During the food product transportation, handling, and storage, the films with a good mechanical strength and ductility are usually needed to withstand external stress. TS and EB are key parameters describing the mechanical properties of the packaging materials [32]. As shown in Figure 4, there was no significant difference in the TS between the CS film and CS-GCNE films (*p* > 0.05). They showed TS values of about 30 MPa, which were equal to commercial films (e.g., polypropylene film) [33]. In contrast, the EB of the films was obviously dependent on the concentration of the GCNE. The EB value increased with the addition of the GCNE (*p* < 0.05), indicating that the crosslink of the film was improved and a tighter network structure was formed. This may be because the GCNE broke the crystal structure of the CS matrix, reduced the rigid structure, and increased the number of flowable polymers [34].

### 3.5. Color and Light Transmittance of the Films

Color is an important characteristic of food packaging film, which affects consumers’ intuitive visual experience during purchase. The color parameters of the films are summarized in Table 1. The CS-GCNE film became less bright and more red with the decreased L and b, and increased a and ΔE. This was mainly owing to the colored substance of the GCNE. However, there was no significant difference in the a value and ΔE value of the CS-GCNE films with different GCNE concentrations, indicating that the red color reached saturation.

**Figure 5:**
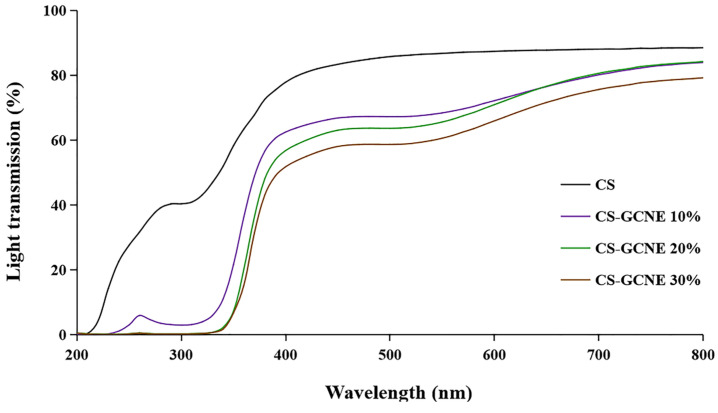
Light transmission at wavelengths ranging from 200 nm to 800 nm of the neat CS and CS-GCNE composite films.

The films used in food packaging are usually required to have high UV-light-blocking properties to protect the food from UV damage. The UV–vis light transmission of the films incorporated with the different GCNE concentrations in 200–800 nm are shown in Figure 5. Overall, the films containing the GCNE showed lower transmittance compared with the neat CS film. It is noteworthy that the decrease of the transmittance of the CS-GCNE films in UV light (280–320 nm) was in the range of 92–99%. The results indicated that the CS-GCNE films had higher light-barrier properties, especially UV-light-blocking properties, than the neat CS film. This was mainly because the GCNE possessed pigments and UV absorbents, such as flavonoids and phenolic compounds.

### 3.6. MR, WA, and WVP of the Films

The MR and WA of the films are related to the water sensitivity of the films, which could influence the packaging film application in a high-humidity environment. The MR and WA of the films are summarized in Table 2. The MR and WA of the CS-GCNE films were obviously lower than that of the CS film (*p* < 0.05). The higher the GCNE content, the lower the MR and WA. The results indicated that the CS-GCNE films had a lower water sensitivity and higher stability than the neat CS film. The GCNE may restrict the hydrogen-bonding interaction between the water and CS molecules, potentially leading to this outcome. A similar result was obtained by Luo et al. [29].

The WVP of the films is related to its efficiency to prevent moisture loss. Ideal food packaging film is usually required with a low WVP to block the water exchange between the food and the external environment [35]. As described in Table 2, the WVP of the films were not influenced after incorporating the GCNE (*p* < 0.05). Overall, the WVP values were low, which would favor maintaining food quality in packaging [29].

### 3.7. Antioxidant Activities of the Films

**Figure 6:**
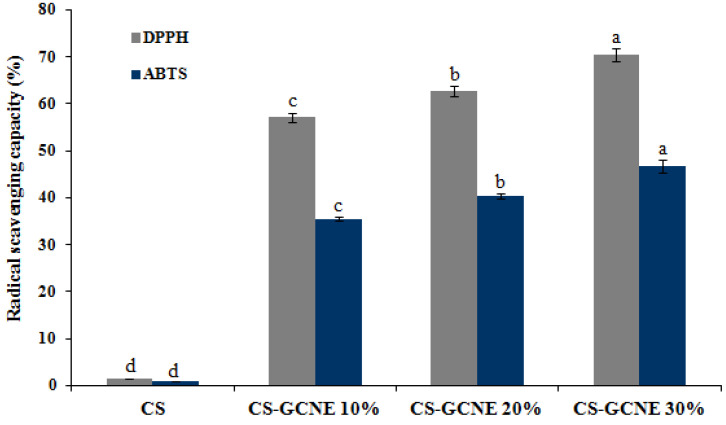
DPPH and ABTS radical scavenging activity of the neat CS and CS-GCNE composite films. Mean values and standard deviation. a–d: different letters indicate significant differences among the films (*p* < 0.05).

The antioxidant activity of the films is related to its efficiency to protect food from oxidation. Analysis of the DPPH and ABTS radical scavenging capacity is widely used as the standard methods to estimate the antioxidant activity of films. As described in Figure 6, the CS film showed little DPPH and ABTS radical scavenging activity, while the CS-GCNE10%, CS-GCNE 20%, and CS-GCNE 30% films exhibited superior DPPH and ABTS radical scavenging activity (DPPH: 57.11 ± 0.99%, 62.71 ± 0.98%, and 70.37 ± 1.33%; ABTS: 35.53 ± 0.50%, 40.36 ± 0.51%, and 46.73 ± 1.39%, respectively). The high DPPH and ABTS radical scavenging activity is most likely attributed to the high phenolic content in the GCNE, which possessed the free-radical capture activity. This result suggested that the incorporation of the GCNE could significantly improve the antioxidant activity, which was in accordance with the previous findings that natural plant extracts rich in phenolic compounds could enhance the antioxidant activity of films [36,37].

### 3.8. Mango Preservation Application

**Figure 7:**
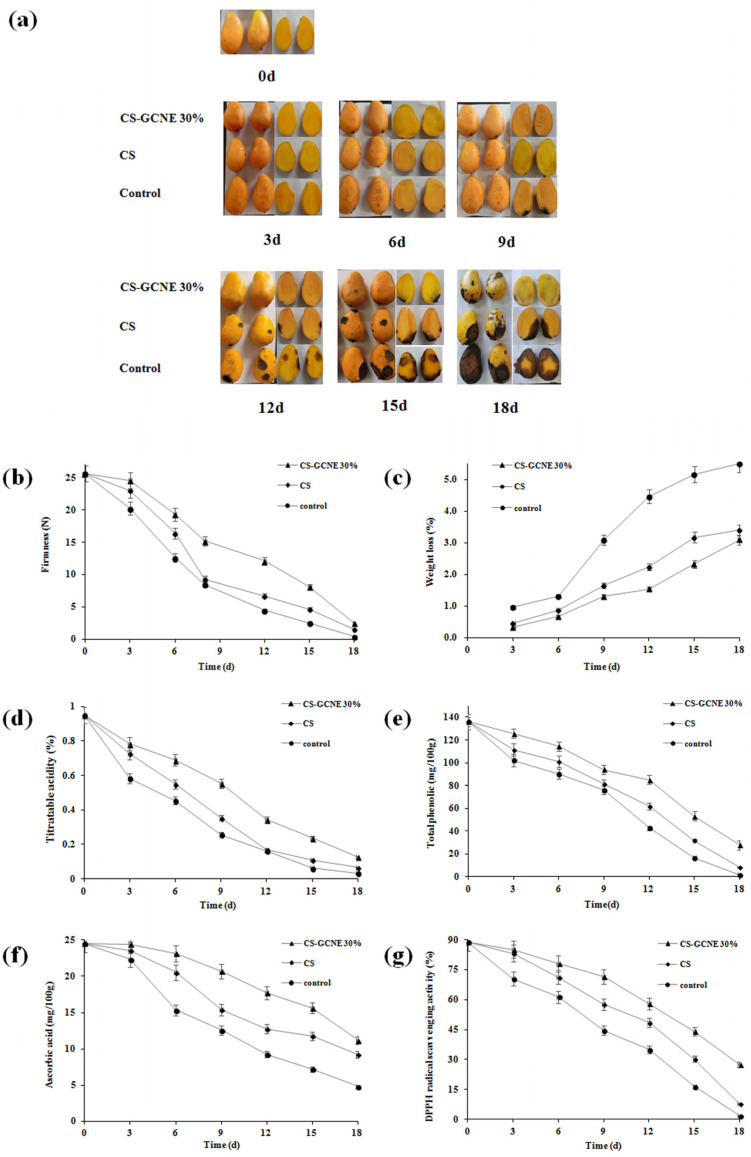
Changes in the appearance (**a**), firmness (**b**), weight loss (**c**), titratable acidity (**d**), total phenolic (**e**), ascorbic acid (**f**), and DPPH radical scavenging activity (**g**) of the control and film-solution-coated mangoes stored at 20 °C for 18 days.

Poor appearance, such as wrinkling, leakage, and decay, usually appear on postharvest fruit surfaces if stored improperly. The edible-film-solution coatings were expected for fruit preservation to maintain the fruit quality. Decay was considered as the most important factor affecting mango appearance [38]. The CS-GCNE 30% film exhibited the highest antioxidant activity. Therefore, it was used as a potential material to preserve mangoes in this study. As illustrated in Figure 7a, there were many differences in the mango appearance in different treatment groups during storage. Compared with the control and CS group, the decay of the mangoes in the CS-GCNE 30% group was slower and to a lesser extent. In the assay, it was not found that the aroma of the mangoes was affected by the composite film.

The firmness of fresh fruit is one of the important indicators of storability, which influences shelf-life. Firmness is a comprehensive performance of a variety of fruit physiological responses, such as respiration, transpiration, and senescence [39]. As illustrated in Figure 7b, the firmness of the mangoes gradually decreased during the entire storage process. The CS-GCNE 30% film coating delayed the firmness decline effectively. This result benefited from the inhibition of the fruit respiration, transpiration, and senescence of the CS-GCNE 30% film coating.

Weight loss in fresh fruit is mainly due to the loss of nutrients and water caused by respiration and transpiration, resulting in pulp weakness and fruit shrinkage [40]. As shown in Figure 7c, the weight loss of the mangoes showed an increased trend during the preservation period. Compared with the control and CS groups, the weight-loss rate of the mangoes in the CS-GCNE 30% group was lower, indicating that the CS-GCNE 30% film coating was effective in preventing weight loss in the mangoes. This was mainly because the CS-GCNE 30% film coating on the mangoes acted as a semipermeable barrier against water vapor and oxygen, which inhibited fruit respiration and transpiration.

The titratable acid content is an important indicator for fruit ripening, which reflects the flavor and storage quality [41]. As illustrated in Figure 7d, the titratable acidity for the CS-GCNE 30% group decreased continuously, and the decreasing trend of the titratable acidity in the CS-GCNE 30% group was slower than in the other groups. This may be owed to the inhibition of the fruit respiration of the CS-GCNE 30% film coating, resulting in nutrient-consumption reduction. The result indicated that the CS-GCNE 30% films, as a food packaging, could maintain fruit flavor and extend the shelf-life.

Phenolic compounds in fruits can resist pathogenic microorganisms [42]. They play an important role in delaying fruit decay. As shown in Figure 7e, the total phenolic content of the mangoes showed a decreased trend during storage. Compared with the control and CS groups, the decrease in the total phenolic content of the mangoes in the CS-GCNE 30% group was lower, indicating the CS-GCNE 30% film coating was effective in maintaining the phenolic compounds of the mangoes. This result was in agreement with the appearance changes.

Ascorbic acid is an important vitamin in fruits. However, it is easily oxidized and decomposed during storage [43]. As shown in Figure 7f, the ascorbic acid content of the mangoes gradually decreased during the preservation period. The CS-GCNE 30% film coating delayed the ascorbic acid content decline effectively. This result benefited from the semipermeable barrier of the CS-GCNE 30% film coating against oxygen, which inhibited the oxidative decomposition of the ascorbic acid of the mangoes.

The antioxidant activity of fruits is related with their antioxidant compounds; for example, phenolic compounds and ascorbic acid. To well-understand the functional property of the CS film by incorporating the GCNE, the antioxidant activity of fruits in different treatment groups was measured. As illustrated in Figure 7g, the DPPH radical scavenging activity of the mangoes showed the same trend in the total phenolic and ascorbic acid contents. Compared with the control and CS groups, the decrease in the DPPH radical scavenging activity of the mangoes in the CS-GCNE 30% group was lower, indicating that the CS-GCNE 30% film coating was beneficial to maintain the antioxidant activity of the mangoes.

## 4. Conclusions

In this research, the multifunctional composite films were successfully developed by incorporating GCNE into the CS matrix. Our results demonstrated that a good interaction was presented among the GCNE and CS through an intermolecular hydrogen bond. The elongation at break, UV-light blocking, and water sensitivity were significantly improved by incorporating the GCNE. Moreover, the multifunctional composite films exhibited an outstanding antioxidant activity. The edible-film-solution coatings could effectively maintain the quality of postharvest mangoes by lowering the changes in the firmness, weight loss, titratable acidity, and total phenolic and ascorbic acids. As the GCNE has a low odor, the CS/GCNE composite coatings showed no negative impact on the fruit’s aroma. The solution form of the coating has the potential to be employed directly, enabling the creation of a protective layer on the fruit’s surface through simple spraying or dipping techniques. It was observed that this coating possesses the convenient attributes of effortless processing, easy application, and low cost in comparison to other works loading other polyphenols [8,9] or tannins in chitosan/MXene (or starch and gelatin)-based films [14,15,16]. Our work emphasizes the potential utilization of CS/GCNE composite films, which exhibit promising qualities as bioactive packaging materials. However, there are some limitations in this work, such as the single fruit variety and the lack of evaluation of the antibacterial activity. To determine the most suitable target fruit and preservation effect, future studies will expand the scope of the test fruit varieties (e.g., berries, drupes, etc.) and assess antimicrobial (e.g., antibacterial, antifungal, etc.) activity. Furthermore, great importance should be placed on the registration and supervision of functional composite films so to guide their reasonable commercialization.

## Figures and Tables

**Table 1:** Color of the films.

Films	L	a	b	∆E
CS	90.35 ± 0.27 ^A^	−0.60 ± 0.60 ^B^	1.55 ± 0.19 ^A^	2.20 ± 0.18 ^C^
CS-GCNE 10%	81.14 ± 0.43 ^B^	4.88 ± 0.44 ^A^	1.90 ± 0.13 ^A^	81.31 ± 0.45 ^A^
CS-GCNE 20%	79.53 ± 0.65 ^C^	4.99 ± 0.40 ^A^	0.35 ± 0.35 ^C^	79.69 ± 0.63 ^B^
CS-GCNE 30%	78.48 ± 1.14 ^C^	5.14 ± 0.70 ^A^	−1.01 ± 0.54 ^C^	78.55 ± 1.12 ^B^

All data are shown as mean ± standard deviation (SD). Different superscripts (A–C) in the same column indicate significant differences among the films (*p* < 0.05).

**Table 2:** Moisture regain (MR), water absorption (WA), and water vapor permeability (WVP) of the films.

Films	MR (%)	WA (%)	WVP (10^−11^ g m^−1^s^−1^Pa^−1^)
CS	16.68 ± 0.81 ^A^	80.65 ± 0.55 ^A^	0.96 ± 0.029 ^A^
CS-GCNE 10%	14.32 ± 0.07 ^B^	71.99 ± 0.64 ^B^	0.94 ± 0.058 ^A^
CS-GCNE 20%	12.56 ± 0.18 ^C^	56.89 ± 0.58 ^C^	0.92 ± 0.031 ^A^
CS-GCNE 30%	10.69 ± 0.18 ^D^	54.74 ± 0.74 ^C^	0.91 ± 0.020 ^A^

All data are shown as mean ± standard deviation (SD). Different superscripts (A–D) in the same column indicate significant differences among the films (*p* < 0.05).

## Data Availability

The data presented in this study are available on request.
